# Causality dilemma: creating a twenty-first century university archive

**DOI:** 10.5195/jmla.2021.1056

**Published:** 2021-01-01

**Authors:** Faye Mazzia, Megan De Armond

**Affiliations:** 1 fmazzia@touro.edu, Assistant Professor and Electronic and Technical Services Librarian, Jay Sexter Library, Touro University Nevada, Henderson, NV; 2 mde_armo@touro.edu, Assistant Professor and Research and Instruction Librarian, Jay Sexter Library, Touro University Nevada, Henderson, NV

## Abstract

For its fifteenth anniversary, the Jay Sexter Library at Touro University Nevada (TUN) sought ways to capture its institutional history by founding an archive. Among many challenges, the library struggled to convince the administration of the importance of an archive. To generate interest in TUN's history, a task force comprising library, executive administration, and advancement staff hosted and recorded a panel event with some of the university's original faculty, staff, and administration. By having this event, new TUN employees were able to experience the shared knowledge of TUN's early days, and the library was able to create and preserve its own institutional history.

## INTRODUCTION

**Figure 1:**
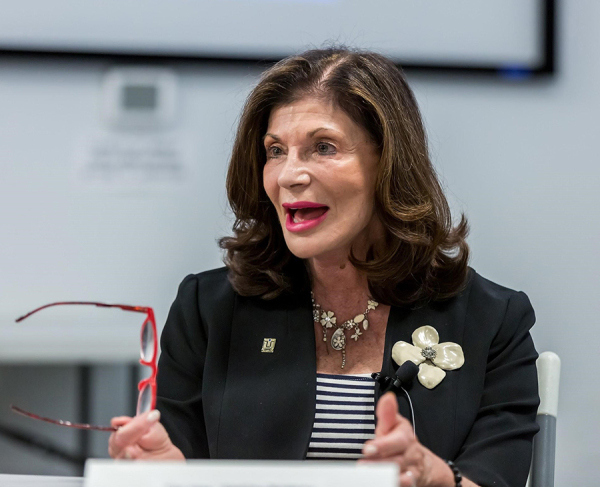
Touro University Western Division Chief Executive Officer and Senior Provost Shelley Berkley

This is the story of how one library with limited resources renewed interest from and engagement with the university community for creating an archive to preserve the institutional history. Touro University Nevada (TUN) established itself in 2004 in the fast-growing city of Henderson as Nevada's first osteopathic medical school and commemorated its 15th anniversary in 2019. TUN is a small (approximately 1,300 students) private institution, part of the Touro University Western Division and the Touro College and University System worldwide. Former Nevada Congressional Representative Shelley Berkley, chief executive officer and senior provost, currently oversees the Touro University Western Division, which comprises TUN and Touro University California (Figure 1). Since its establishment, the programs offered at TUN have expanded, some doubling in size and others moving from master's to doctoral level [1]. As librarians of the TUN Jay Sexter Library (JSL), the authors recognized the importance of preserving and documenting the 15th anniversary not only for our faculty, staff, students, and alumni, but also for the Las Vegas Valley and Nevada as a state.

Photo credit: Mona Shield-Payne.

The only university archive for the Touro College and University System resides in New York, which houses the collection of Bernard Lander, the founder of Touro College [2]. The archive in New York has no holdings connected to TUN, and as far as we are aware, Touro University California does not have an archive. TUN administration and staff have discussed and planned the founding of an archive throughout the years. Still, the archive did not materialize, and priorities shifted toward planning for the future rather than preserving the past. However, the hiring of new library staff in the previous two years revived the idea of establishing an archive.

## CHALLENGES

When TUN library personnel revived the idea of an archive in 2018, we faced a myriad of interconnected challenges. Without a record of the past, preservation and documentation are a challenge. For example, the earliest capture of the TUN's website in November 2009 mentions TUN's fifth anniversary and some “great events to celebrate” [3]. We are not sure what those events were. Those who would know have since moved on or simply do not remember. Instead of dwelling on what was lost in those initial years, we shifted our focus to preserving what is on-hand now.

Though many employees have passed through TUN, several original members of TUN remain. Unfortunately, those employees with collections pertaining to the history of TUN have been reluctant to donate their collections. Several months prior, JSL tried a different approach to establishing an archive. The library surveyed all TUN departments to determine what types of records each department kept, if they had a record management policy in place, and if they would be interested in transferring their files or materials to the custody of the library. The library used the SurveyMonkey platform and distributed the survey link via email to thirty-eight TUN department heads, directors, prominent faculty, and staff, as well as long-standing employees. A follow-up email was sent to reiterate the relevance and meaning of the responses. Of the eight people who completed the survey, only two expressed a willingness to donate materials to a TUN archive, one of whom retired immediately afterward.

Justifying the purchase of archival supplies was another challenge. The few physical accessions that had already been acquired remained in their original folders and binders. They were inventoried, but no finding aid was created. When library personnel proposed a budget for archival materials, it was often suggested that they “just digitize everything.” A common misunderstanding is that digitization and preservation are the same, but, alas, digitization is not preservation, which poses another archival concern, digital preservation. Caplan points out that digital preservation involves several activities that ensure the availability, identity, understandability, fixity, authenticity, viability, and renderability of digital information [4]. Our struggle is not only with material acquisition, but also with having the right tools, skills, and software for digital preservation. In Henderson's article on copyright, she discusses the “archival function” of libraries and the difficulty of accomplishing this function with rapid technological change: the disappearance of information after a short time, the fragility of digital bits, and the short life of hardware and software [5].

Despite these hurdles, we see the need to showcase TUN's roots, history, and connection to the broader community. TUN is the first osteopathic medical school in the state of Nevada, and we believe this identity is enough to justify the formation of an archive. Theimer simply states that archives are “the repository for the historical records of its parent organization” [6]. She later emphasizes the archivist's role in preserving the meaning and relevance of context [6]. Just as a resume provides background information and credentials, an institutional archive shows outsiders the university's legitimacy or, at the very least, sufficient settlement to afford reflection. An archive promises permanence to students and transparency to stakeholders and the community.

## CAUSALITY DILEMMA

The causality dilemma, more commonly known as the chicken or the egg paradox, characterizes situations in which it is difficult to determine which of several events are causes and which are effects. One can summarize JSL's archive effort in the following causality dilemma: we do not have an archive because no one will donate their collections to us, and no one will donate their collections because we do not have an existing archive. Just as it is unrealistic to solve one clause without solving them both, it seemed impossible for JSL to make any tangible headway on the project.

When faced with these sorts of dilemmas, one approach is to make assumptions about each clause or party involved and act on those assumptions.

First, though we lacked many resources—space, experienced staff, time, archival materials, and budget—we assumed that the main reason that we did not have an archive established was because of a lack of relevant donations. We isolated this one reason and collaborated on a solution, which was to create something of our own that captured the TUN experience and community to archive for the future instead of trying to solicit others' materials.

Second, we assumed the common reason why long-standing employees did not want to give their papers to the library was for privacy reasons. To combat this, instead of asking for a person's entire collection and expecting them to trust us with potentially personal information, we opted for a loosely structured interview that would allow the interviewee control over what they did and did not want to share.

Combining these two assumptions and solutions, we decided to host a panel event. Participants would feel as if they had control over what information they wanted to share, and we would get something of archival value, namely, firsthand accounts of TUN's founding. To increase exposure and interest in the archive, we chose to make the TUN Pioneers Panel Event open to the entire university. The plan was simple: host the event, record it, transcribe the stories, and then digitally preserve the recording and transcription for the foreseeable future.

## TOURO UNIVERSITY NEVADA PIONEERS PANEL EVENT

The approach of TUN's fifteenth anniversary was the perfect opportunity to host our panel event. A planning group formed quickly and involved members from JSL, Executive Administration, and the Department of Advancement. For panelists, we invited staff and faculty who were with the institution since doors opened in 2004—ten of whom were current employees, one a recent retiree, and the original provost—to participate in oral histories. Of those twelve invitees, six accepted.

**Figure 2:**
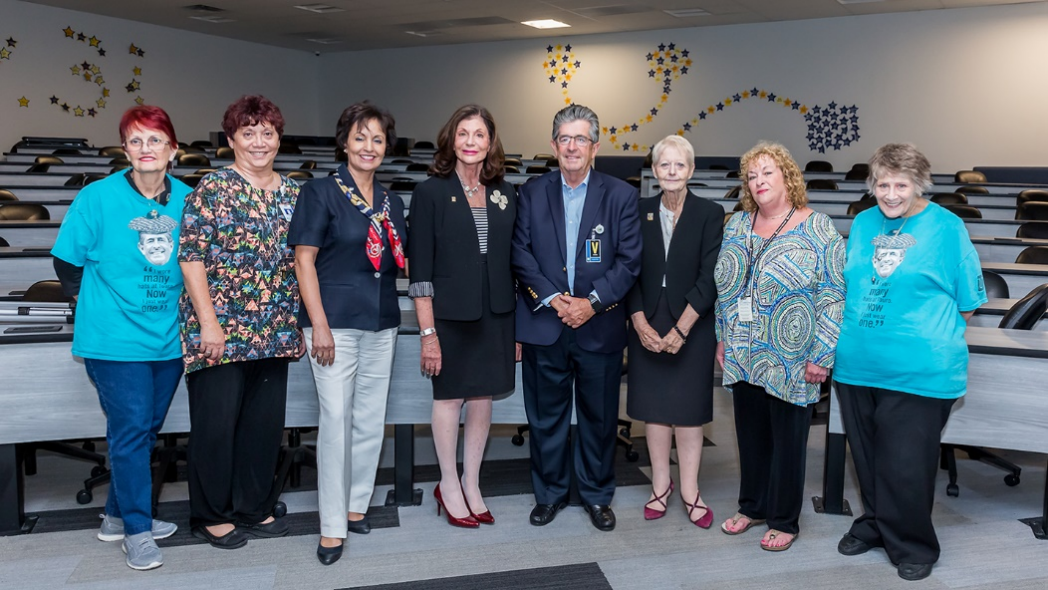
The Touro University Nevada (TUN) Pioneers panelists

Our panel was hosted and moderated by Chief Executive Officer and Senior Provost Berkley. Executive Administration provided the catering, and the Department of Advancement video-recorded the event, live-streamed it on Facebook, and hired a professional photographer to capture highlights of the event. The theme of the panel was “The Early Days,” and questions included: “How did you end up at Touro?”; “What are some challenges you faced?”; and “What are some of your earliest memories?” Each participant had time to answer questions and share a few anecdotes (Figure 2). The one-hour event was well received and attended by many TUN faculty and staff.

From left to right: Dee Anne Culmone, Terry Else, Amina Sadik, Shelley Berkley, Michael Harter, June Boyko, Lisa Ross, Jackie Boiman.

Photo credit: Mona Shield-Payne.

## REFLECTION AND LESSONS LEARNED

A common theme discussed during the TUN Pioneers Panel Event was the daring and enterprising spirit that prevailed during those beginning days. Panelists reflected on the challenges that were overcome with good humor, negotiation, and unconventional approaches: office furniture was distributed to faculty and staff via a lottery; there were no telephones; and the first incoming class toured the campus wearing safety helmets. The limitations TUN faced in its early days—no space, few resources, little money—ring true to the challenges JSL faces today in creating an archive.

The key to success for the founders of TUN was that they relied heavily on personnel. Founder Jay Sexter and President Michael Harter had handpicked a buoyant, flexible, and ambitious team that was committed to building TUN. The TUN Pioneers Panel Event was not only valuable because it was a moment to share and strengthen community ties, but it also gave JSL personnel clues on how to create something of their own out of nothing.

## NEXT STEPS

The positive reception of the TUN Pioneers Panel Event gave JSL librarians hope to pursue preserving institutional history in the form of oral histories. JSL will focus on one-on-one oral history interviews with long-standing faculty and staff, looking to best practices prior to scheduling these interviews, which we plan to do in-person when possible. We plan to connect with the Medical Library Association's Oral History Committee and History of the Health Sciences Caucus to look for guidance and support. We also plan to connect with other universities of the same size with an existing archive to get ideas for ways to strategize. We are exploring having an outside expert come to speak with us about setting up an archive as well.

We believe TUN's history matters and hope to instill confidence in our colleagues that the library could be a viable home for that history. With the video recording saved in multiple locations and the transcription of the panel nearly complete, JSL librarians are satisfied with the byproducts of this endeavor.
